# Mechanical Analysis of Feeding Behavior in the Extinct “Terror Bird” *Andalgalornis steulleti* (Gruiformes: Phorusrhacidae)

**DOI:** 10.1371/journal.pone.0011856

**Published:** 2010-08-18

**Authors:** Federico J. Degrange, Claudia P. Tambussi, Karen Moreno, Lawrence M. Witmer, Stephen Wroe

**Affiliations:** 1 CONICET - División Paleontología Vertebrados, Facultad de Ciencias Naturales y Museo, Museo de La Plata, Universidad Nacional de La Plata, La Plata, Argentina; 2 Laboratorio de Paleontología, Instituto de Geociencias, Universidad Austral de Chile, Valdivia, Chile; 3 Evolution and Ecology Research Centre, School of Biological, Earth and Environmental Sciences, University of New South Wales, Sydney, Australia; 4 Department of Biomedical Sciences, College of Osteopathic Medicine, Ohio University, Athens, Ohio, United States of America; Zoological Society of London, United Kingdom

## Abstract

The South American phorusrhacid bird radiation comprised at least 18 species of small to gigantic terrestrial predators for which there are no close modern analogs. Here we perform functional analyses of the skull of the medium-sized (∼40 kg) patagornithine phorusrhacid *Andalgalornis steulleti* (upper Miocene–lower Pliocene, Andalgalá Formation, Catamarca, Argentina) to assess its mechanical performance in a comparative context. Based on computed tomographic (CT) scanning and morphological analysis, the skull of *Andalgalornis steulleti* is interpreted as showing features reflecting loss of intracranial immobility. Discrete anatomical attributes permitting such cranial kinesis are widespread phorusrhacids outgroups, but this is the first clear evidence of loss of cranial kinesis in a gruiform bird and may be among the best documented cases among all birds. This apomorphic loss is interpreted as an adaptation for enhanced craniofacial rigidity, particularly with regard to sagittal loading. We apply a Finite Element approach to a three-dimensional (3D) model of the skull. Based on regression analysis we estimate the bite force of *Andalgalornis* at the bill tip to be 133 N. Relative to results obtained from Finite Element Analysis of one of its closest living relatives (seriema) and a large predatory bird (eagle), the phorusrhacid's skull shows relatively high stress under lateral loadings, but low stress where force is applied dorsoventrally (sagittally) and in “pullback” simulations. Given the relative weakness of the skull mediolaterally, it seems unlikely that *Andalgalornis* engaged in potentially risky behaviors that involved subduing large, struggling prey with its beak. We suggest that it either consumed smaller prey that could be killed and consumed more safely (e.g., swallowed whole) or that it used multiple well-targeted sagittal strikes with the beak in a repetitive attack-and-retreat strategy.

## Introduction

Phorusrhacids were a predominantly South American radiation of gruiform birds known from middle Paleocene to lower Pleistocene deposits [1], [2] and most closely related to seriemas (Cariamidae) among extant birds [3]-[6]. Their often gigantic body sizes, large skulls, and carnivorous lifestyles have resulted in phorusrhacids being popularly termed “terror birds.” *Andalgalornis steulleti* [7], from the upper Miocene–lower Pliocene (≈6 million years ago) of Argentina, was a medium-sized patagornithine phorusrhacid of about 40 kg body mass, 1.4 m height, and 370 mm total skull length. All clades of phorusrhacids (Patagornithinae, Phorusrhacinae, Brontornithinae, Psilopterinae, Mesembriornithinae; [5]) are considered to be flightless ground predators or scavengers and ranged from 0.9 to 3.0 m in height [1], [8]. They are often regarded as apex predators that dominated South American Tertiary environments in the absence of carnivorous placental mammals [1]. However, phorusrhacids were largely contemporaneous with the carnivorous borhyaenid marsupial radiation, which also included some large to gigantic species [9]. To date, hypotheses of feeding ecology in terror birds have been based mostly on the presence of large skulls with hooked beaks and have yet to be supported by appropriate biomechanical studies. Here we perform a biomechanical analysis of the skull of *Andalgalornis steulleti* (FMNH P14357) in a comparative context, using comparative anatomy and a powerful engineering tool, Finite Element Analysis (FEA), to predict the mechanical behavior of its skull.

## Results

### Divergent skull morphology and loss of cranial kinesis

The nature of the connections between the skull bones is important to the analysis of mechanical behavior, because these connections influence the relative movement of areas of the skull (i.e., cranial kinesis) [10] and hence the distribution of stress and strain [11], [12]. Holliday and Witmer [10] recently proposed four criteria for inferences of intracranial mobility in extinct taxa. Extant neornithine birds (including seriemas) generally meet all four criteria, and in the vast majority of extant birds part or all of upper bill can be elevated and depressed relative to the braincase, although these movements have been analyzed mechanically in only a few taxa [13], [14], [15]. The fact that phorusrhacids are phylogenetically embedded within Neornithes indicates that they evolved from fully kinetic ancestors. The skull of *Andalgalornis*, however, shows some features suggesting loss of intracranial immobility. We assessed the detailed morphology of these features in *Andalgalornis* and some other phorusrhacids using CT scanning, supplementing these findings with observations of other taxa.

**Figure 1:**
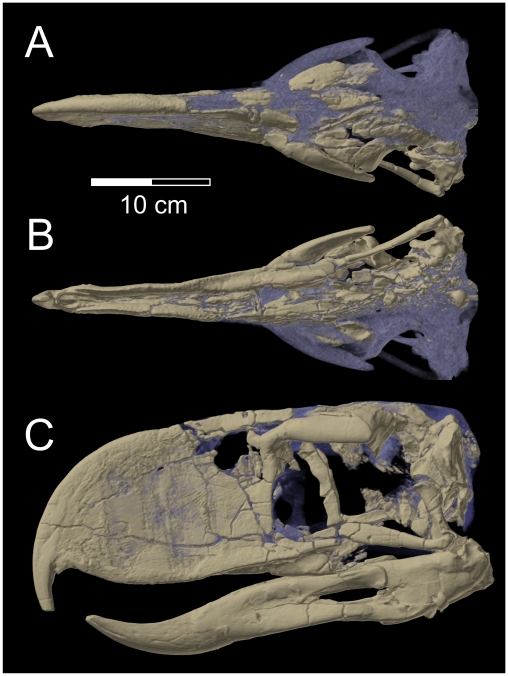
Skull of *Andalgalornis steulleti* (FMNH P1435). A, dorsal view, B, ventral view, and C, left lateral view, based on volume rendering of CT scan data. Fossil bone is shown in light brown, and rock matrix and plaster restoration are shown in grey.

A key element of extant avian kinesis is the transformation of bony connections that are immobile in nonavian outgroups into thinned “flexion zones” that permit bending movements [10], [13]–[17]. These flexion zones, satisfying the criterion of “permissive kinematic linkages” [10], typically are found in three locations in extant birds where the bones become strongly dorsoventrally flattened, permitting elevation/depression movements of the upper jaw segment: (1) the craniofacial hinge (within or between the premaxillae, nasals, and frontal), (2) the palatine bones, and (3), the jugal bar. Although the available skull of *Andalgalornis* (FMNH P14357) is somewhat damaged in the region corresponding to the craniofacial flexion zone of other birds, CT scans reveal that enough structure is preserved to show remarkably thick bone in this region relative to that present in extant seriemas or other kinetic birds. Moreover, the lacrimal bone is fused rostrally with the upper bill (nasal and maxillary bones) and caudally with the frontal bone, effectively spanning and further restricting movement at the craniofacial flexion zone. Likewise, the flexion zone in the palatine bone clearly has been transformed into a rigid structure in that the bone has become thickened and folded into a U-shaped conformation. Finally, the flexion zone in the jugal bar has also been apomorphically transformed into a thickened beam that is mediolaterally elliptical rather than dorsoventrally flattened. The absence of any of these permissive kinematic linkages individually would be enough to limit or prevent kinesis, but the absence of all three clearly reflects enhanced rigidity and no movement of the upper bill relative to the braincase. Additionally, the lacrimal bone bears an expanded descending ramus that is firmly sutured to the jugal bar, further locking these units together (Figure 1, Text S1, Figure S1), rather than being kinematically separated as in birds retaining cranial kinesis. Other phorusrhacids appear to show a mosaic of secondarily akinetic attributes, suggesting that, although they all seem to have had reduced or no kinetic mobility, they vary in the extent to which they have rigidified the former flexion zones. For example, specimens of most of the taxa studied reveal the thickened and folded palatines, but some of the smaller-bodied taxa (e.g., psilopterines) may have retained craniofacial and jugal flexion zones closer to the plesiomorphic condition. But all available evidence suggests that large-bodied taxa, such as other patagornithines (e.g., *Andrewsornis*, *Patagornis*) and the truly gigantic phorusrhacines (e.g., *Kelenken*, *Devincenzia*), resembled *Andalgalornis* in transforming all three flexion zones into thickened, reinforced, and immobile junctions.

Zusi [17] regarded cranial kinesis as a universal feature of all birds, but various birds have been considered as akinetic (e.g., ratites [18], penguins [19], collies [20], toucans [21], hawfinch [22]). This is the first time that akinesis of a gruiform has been reported, and may be among the best documented cases forwarded for any bird based on anatomical grounds. It is likely that more morphological analyses like the present one will reveal more cases of secondarily reduced or lost kinetic ability in other birds.

### Bite force

Published *in vivo* bite force data for birds is largely restricted to Passeriformes [23], [24], a derived clade of generally small-bodied animals. These studies show that bite force is related to skull morphology and geometry, as well as the capacity of contraction of the jaw muscles [23,25,26). In Galápagos finches, beak size and especially head width are strongly correlated with bite force, and head size closely correlates with jaw muscle dimensions [27], [28]. Passeriforms are obviously a poor model for the estimation of bite forces in phorusrhacids, due to differences not only in phylogeny but also in skull size and morphology.

To predict bite force in *Andalgalornis*, we constructed a regression line (R = 0.8123; R^2^ = 0.6598; p = 0.0000) using three different data sources: published avian bite force data [23], our own bite force data obtained with a transducer designed to measure forces *in vivo*, and body mass data [23], [29], [30] (see Materials and Methodbelow and Table S1). Based on regression analysis (Figure S2) including the seriema (50 Newtons) and eagle (50 N), we estimate the bite force of *Andalgalornis* as 133 N at the bill tip.

### Finite element analysis

Finite Element Analysis (FEA) is an engineering approach initially developed to predict stress/strain distributions in man-made objects with relatively simple geometry. With advances in computer technology FEA has emerged as a powerful tool in the investigation of mechanical behavior in complex biological structures, its nondestructive nature rendering it of particular value in paleontological studies [31], [32].

The *Andalgalornis* skull model recorded lower mean brick stress (load per unit area) than either *Cariama* or *Haliaeetus* under three extrinsic (i.e., movements of the prey in relation to the head [33]) loads (See Materials and Method and Table 1). We consider it likely that the skull of *Andalgalornis* could have supported larger bite loads than the one tested in this study (133 N), although validation would be required to firmly establish this. In a comparative context, the mechanical behavior of *Andalgalornis* differs further in that mean “brick” element stress values (Von Mises, VM) for the lateral shake simulation were four times greater than those recorded for pullback values. The pullback loading generated the least stress in each of the three skulls. In both *Cariama* and *Haliaeetus*, mean stresses under lateral-shake and normal bite loadings were similar. For *Andalgalornis*, however, the lateral-shake loading induced mean brick stresses that were double those generated by the normal bite.

**Table 1:** Mean brick element stresses (von Misses, VM) in solved FE models under three load cases; L = lateral shake, N = normal bite, P = pullback.

	*Andalgalornis*	*Haliaeetus*	*Cariama*
Element number	1,080,137	860,757	775,698
Lateral shake	1.028	2.272	3.285
Normal Bite	0.570	2.412	3.235
Pullback	0.234	0.678	0.833
L/N ratio	1.803	0.941	1.015
L/P ratio	4.393	3.351	3.943

**Figure 2:**
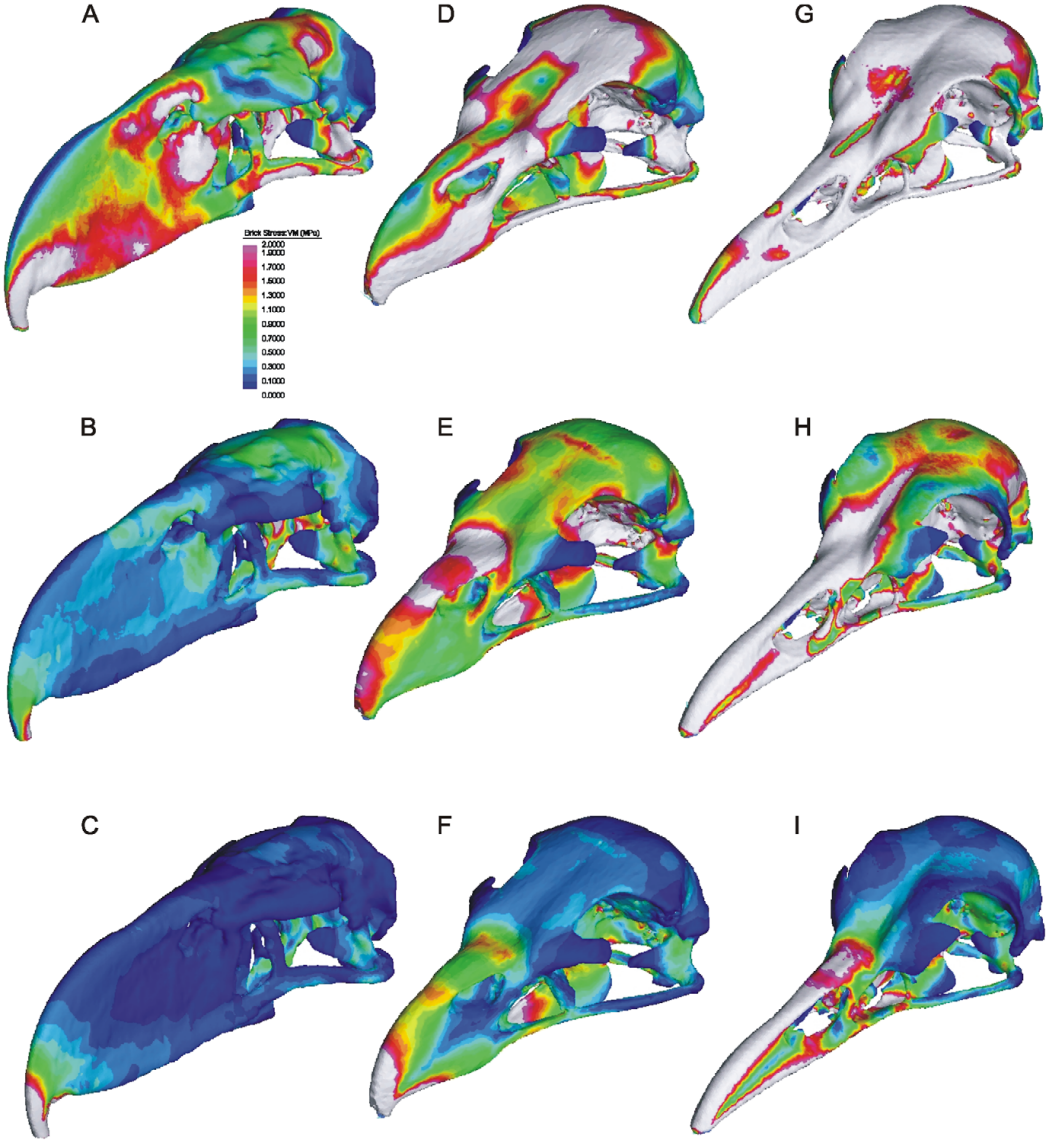
Stress (Von Mises) distribution of FE models. (A–C) *Andalgalornis steulleti*, (D–F) *Haliaeetus albicilla* and (G–I) *Cariama cristata* under three loads cases: (A, D, G) lateral shake, (B, E, H) Normal Bite and (C, F, I) Pullback. VM = Von Mises; MPa, mega pascals. White areas indicate VM exceeds the scale maximum (2 MP) at those areas.

In all three load cases applied to *Andalgalornis*, three areas concentrated the highest stress: the bill tip (i.e., the hook), the occipital condyle, and the contact between the palatines and pterygoids with the interorbital septum (Figure 2A, B, C). Skull stress was clearly highest under the lateral-shake simulation (Figure 2A). The same is true for *Cariama*, but, in the case of the eagle, the stress was higher in the normal bite than in the lateral-shake extrinsic load. Moreover, in *Andalgalornis*, the stress was highest in the ventrolateral rims of the beak (the tomia), the dorsal surface of the nasal bones, the rostral edge of the antorbital fenestra (below the naris), the body of the quadrate bone, in the temporal fossa, and, the lower margin of the zygomatic arch (jugal bar).

## Discussion

Our comparative FE analyses show that the skull of *Andalgalornis* was best optimized to resist rostrocaudally and dorsoventrally directed loads, but less well-adapted to resist laterally directed loadings. VM stress values obtained for *Andalgalornis* are consistent with a very high vertical/transverse index of the rostrum. Moreover, as noted above, the plesiomorphically dorsoventrally flattened flexion zones have been transformed into thickened bony connections that are reoriented into more vertically disposed, transversely compressed bars that would resist sagittal loads well but be relatively weak with regard to lateral loads. In birds with a vertical/transverse index of approximately 1.0 (i.e., beaks as wide as high), such as *Cariama* or *Haliaeetus*, the skull would be expected to be equally resistant to forces directed laterally and vertically, which is consistent with our results. Likewise, both seriema and eagle retain the dorsoventrally flattened flexion zones required for kinesis.

The loss of cranial kinesis in *Andalgalornis* and other large-bodied phorusrhacids is a remarkable derived character, but the interpretation of this loss is complicated by persistent uncertainties about the functional role of cranial kinesis [10], [34]. Recent FE studies have revealed that the soft tissues within sutures may function as shock absorbers, acting to lower potentially damaging tensile stress and that loss of kinesis enhances skull rigidity and robusticity, but potentially at the cost of local increases in strains [11], [12], [35]. Thus, we consider it probable that the high stresses recorded in the interorbital septum in our FE model of *Andalgalornis* are artifactual, because the model did not incorporate sutures between the palatines, pterygoids, and the septum. This region may act as a stress releaser, compensating at least in part for loss of the kinetic system.

A carnivorous lifestyle for phorusrhacids has been widely assumed [1], [3], [5], [36], but more detailed hypotheses of predatory behavior have never been adequately tested. Previous reconstruction and biomechanical analysis of the jaw adductor musculature of *Andalgalornis* [37] shows that the jaw apparatus is optimized for strength at the expense of speed, and again, our discovery of loss of cranial kinesis is consistent with increased skull rigidity. Any adaptation that results in increased stiffness of the jaw apparatus, such as transformation of mobile flexion zones into rigid struts, will result in some increase in maximum theoretical bite force [31], [38]. On the other hand, there may be complex trade-offs between safety factors, the presence or absence of kinesis, and the bone and muscle masses required to achieve any given performance limits. For example, a stiffer and more brittle structure (as in akinesis) may result in higher yield points and some reduction in the muscle mass required to achieve a given bite force, but it may also limit the response time available to modify behavior and avoid catastrophic failure where an organism bites into unexpectedly resistant materials [31]. Regarding the loss of cranial kinesis, a net consequence might be that more bone or bone of greater density (i.e., greater bone mass), might be needed to maintain effective safety margins. In clearly akinetic phorusrhacids such as patagornithines and phorusrhacines, the requisite increased bone mass was presumably tolerated because their large body masses precluded flight and hence the premium on ultra-light structures. Future FE based analyses could aid in the quantification of such interrelated factors.

Because most carnivorous birds use the hook on the tip of their beak to hold and kill the prey (e.g. falcons [39]), all bite loads were applied at the beak tip in our FE models. The functional significance of a 133 N bite force for a 40 kg bird is difficult to interpret because of the paucity of comparative bite-force data. The recording of actual bite forces from living birds is restricted to a few passeriforms, and there are no comparable extant carnivorous bipeds. Certainly for its size, our estimate of bite force in *Andalgalornis* is relatively weak compared to predictions generated for anterior bites in carnivorous mammals. For example, the 7.1 kg jaguarondi, *Felis yagouaroundi*, bites at 127 N [40], the fox *Pseudalopex griseus* (5.9 kg) bites at 131 N, and the mustelid *Lutra longicauda* (7.8 kg) bites at 129 N [41]. This is at least in part explained by the comparatively longer distance between the bite point and the jaw joint (outlever) in the terror bird. However, we also note that mean VM stress computed in our modeling of *Andalgalornis* is much lower than that calculated for *Cariama* or *Haliaeetus*. This may indicate safety factors are unusually high in *Andalgalornis* or that our predicted bite force for the phorusrhacid is an underestimate, but differences in size between the specimens makes these questions difficult to evaluate. Moreover, our modeling does not attempt to simulate the influence of jaw head flexor muscles, which may act to amplify total recorded bite forces [32].

Alvarenga and Höfling [5] suggested that the beak morphology of phorusrhacids allowed them to hunt in regions with high vegetation and to catch small animals hiding under stones or fallen tree trunks. Additionally, biomechanical analyses of locomotor capabilities [42], [43] suggest that speed and maneuverability in a phorusrhacid of the size and proportions of *Andalgalornis* would be comparable to those of an ostrich. Our biomechanical analyses reveal that if *Andalgalornis* used its beak in the dispatch of relatively large prey, then it must have been applied with considerable precision in order to avoid sustaining high lateral loads. We suggest that *Andalgalornis* consumed relatively small prey (i.e., smaller than itself) that could be killed and consumed more safely. If *Andalgalornis* did take large prey, then it most likely applied multiple well-targeted strikes in a repetitive attack-and-retreat strategy. Restraining struggling prey with their feet also was potentially an option, despite the absence of sharp talons.

## Materials and Methods

### CT scanning

The skulls of the patagornithines *Andalgalornis steulleti* ( =  *A. ferox* [44]; FMNH P14357) and *Andrewsornis abbotti* (FMNH P13417) and of the psilopterine *Psilopterus lemoinei* (FMNH P13257) were scanned at O'Bleness Memorial Hospital in Athens, Ohio, using a General Electric (GE) LightSpeed Ultra Multislice CT scanner equipped with the Extended Hounsfield option. Specimens were scanned helically at a slice thickness of 625 µm, 120 kV, and 200–300 mA. Moreover, another skull of *Psilopterus lemoinei* (AMNH 9257) and a skull of the extant red-legged seriema (*Cariama cristata*, FMNH 105635) were scanned at the Ohio University MicroCT Scanning Facility (OUμCT) on a GE eXplore Locus MicroCT Scanner at a slice thicknesses of 92 µm and 45 µm, 80 kV, and 500 µA. Data were output from the scanner in DICOM format and then imported into 4.1.2 (Mercury-TGS, Chelmsford, MA) for viewing, analysis, and visualization. The CT scan data were analyzed to assess the internal architecture of bony regions relevant to assessments of cranial kinesis, which were supplemented with gross observation of the skulls of these specimens and others.

### Body mass estimation

The body mass estimate of 40 kg for *Andalgalornis* was made following previously published methodology [45] and submersion of scale plastic models [43, unpublished data].

### Bite force

Bite force data were obtained *in vivo* from captive birds: an adult *Cariama cristata* at the Zoological Garden of La Plata, Argentina, and an adult black-chested eagle (*Geranoaetus melanoleucus*) from the Horco Molle Reserve, Argentina. *G. melanoleucus* is similar in size to *H. albicilla*, and its bite force was used as a proxy in the FEA of *H. albicilla*. For each animal, multiple bites at the bill tip were recorded with the transducer, and we use the maximum bite force in the analysis. The safety standards related to health, hygienic, and dietary plans were followed for the proper management of wildlife, issued by the application and enforcement authority (Dirección de Fauna of Ministerio de Asuntos Agrarios of Buenos Aires Province, Argentina) within the framework of the Provincial Law Decree 12.238/98 and 2308/01. Measurements followed a protocol made in conjunction with the authorities of the establishment which involved measurements taken in the morning before the daily intake, no admission to the environments in which birds are housed and, in all cases, with the assistance of the zookeeper for each animal.

### Finite element analysis

Finite element analysis (FEA) has emerged as a powerful tool in the prediction of biomechanical behavior among both extant and extinct animals [38], [46]-[52]. The protocols implemented in the present study largely follow recently developed methodologies [31], [38], [51], [52].

**Figure 3:**
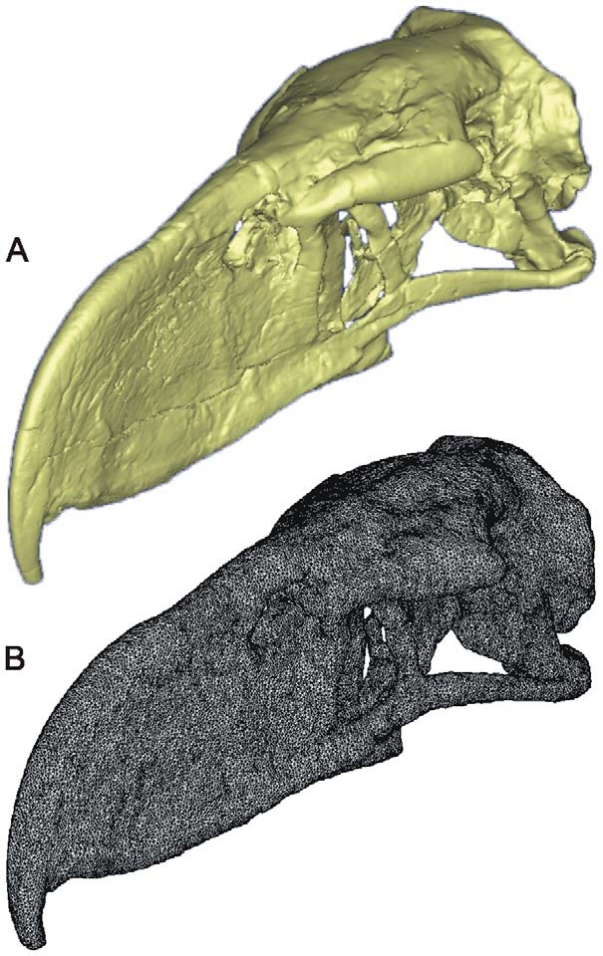
*Andalgalornis steulleti* 3D models. (A) Solid model and (B) 1080137 brick homogeneous finite element model.

Homogeneous models (assuming constant material properties throughout) were constructed using the CT data for *Andalgalornis* (FMNH P14357) with segmentation performed using Mimics (v. 11.01) software (see Figure 3A). A similar procedure was used for the red-legged seriema (*Cariama cristata*, FMNH 105635) and the White-tailed Eagle (*Haliaeetus albicilla*, from the University of Austin Digital Morphology Web site: http://www.digimorph.org). *Cariama cristata* was chosen for comparison because of its close phylogenetic relationships with phorusrhacids and *Haliaeetus albicilla* because of its predatory lifestyle.

Solid meshing was performed with Strand7 Finite Element software (v. 2.3) (Figure 3B). Models were assembled using 3D low-order four-noded tetrahedral “brick” elements (tet4). In the original surface mesh, maximum and minimum triangle edge lengths were kept at a 1∶3 ratio (0.1 geometric error) to minimize differences between triangle dimensions, which can lead to major discrepancies in brick element size in the final solid and introduce artifacts. Brick elements were assigned a single set of material properties as applied in previous studies [31], [32], (Young's modulus of Elasticity [Y] = 27.0 GPa; Poisson's ratio  = 0.4; density  = 2.19085 T/mm^3^).

Models were restrained to prevent free body motion. Point constraints (restricted to single nodes) can produce pronounced artifacts and inaccurate results [48]. For each loading we have applied more realistic constraints by positioning them within frameworks of rigid links at the occipital condyle as well as at the tip of the beak to more broadly distribute forces in accordance with previously published methods [32]. Statistical analyses were performed using a customized program written in RGUI (by K. Moreno).

Two kinds of load cases were arranged and solved: simulation of muscle forces generated by the jaw adductors and head flexors (intrinsic) and simulation of prey movement relative to the predator, or movement by the predator relative to the prey(extrinsic). To study biomechanical performance, we simulated dispatch (killing) forces: rostrocaudal (“pullback”), as well as dorsoventral and lateral shaking. To perform the simulations, we applied the bite force (intrinsic) obtained for each taxon as a load to the distal end of the premaxillary bone (i.e., the hook), with the occipital condyle fixed. Muscles themselves were not reconstructed. Relative mechanical performance was assessed on the basis of mean Von Mises stress for the skull, as well as visual output from the post-processing software.

It is important to note that in the absence of validation our results can only be considered in a comparative context, and, because the models are homogeneous, our approach addresses the influence of geometry, but not differences in the distribution of material properties. Thus, while our results provide insight into the influence of different geometries on the distribution of stress in a relative sense, such findings cannot be considered as absolute performance values [31], [32].

The three taxa considered in this study span a considerable range of cranial dimensions and this raises the issue of scaling. Bite forces have effectively been scaled on the basis of regression and these same forces have been applied as extrinsic forces. In comparative linear-static analyses such as those performed here, scaling will influence the absolute magnitudes of recorded stresses, but not how they are distributed. For example, scaling will not affect interpretations of whether the cranium of *Andalgalornis* is relatively better or less well-adapted to resist lateral vs dorsoventrally directed forces (the ratios will remain the same). Thus, our interpretations are based on proportional rather than absolute differences in mean stress values and distributions.

## Supporting Information

Figure S1 Skull of *Andalgalornis steulleti* (FMNH P1435). Left lateral view (volume rendering of CT scan data) with slice planes (A-B) displaying the hollow beak cavity.
(3.96 MB TIF)

Figure S2 Log bite force in birds plotted against log body mass. Passeriforms in blue (dark blue: Estrildidae, sky blue: Fringillidae), Rheidae in green, Accipitridae in red, Cathartidae in rose and Cariamidae in black. See Table S1 for raw data.
(3.39 MB TIF)

Table S1 Bite force and body mass table.
(0.07 MB DOC)

Text S1 Andalgalornis' fossil.
(0.03 MB DOC)

## References

[1] Tambussi CP, Noriega JI, Arratia G (1996). Summary of the Avian Fossil Record from Southern South America.. Contributions of the southern south America to vertebrate paleontology, Müncher Geowissenschaftliche Abhandlungen.

[2] Tambussi C, Ubilla M, Perea D (1999). The youngest large carnassial bird (Phorusrhacidae, Phorusrhacinae) from South America (Pliocene-Early Pleistocene of Uruguay).. J Vert Paleontol.

[3] Andrews C (1899). On the extinct birds of Patagonia.. Trans Zool Soc London.

[4] Livezey BC (1998). A phylogenetic analysis of the Gruiformes (Aves) based on morphological characters, with an emphasis on the rails (Rallidae).. Tran R Soc London.

[5] Alvarenga HMF, Höfling E (2003). Systematic revision of the Phorusrhacidae (Aves: Ralliformes).. Papeis Avulsos de Zoologia.

[6] Mayr G (2002). A new specimen of *Salmila robusta* (Aves: Gruiformes: Salmilidae n. fam.) from the Middle Eocene of Messel.. Paläontologische Zeitschrift.

[7] Kraglievich L (1931). Contribución al conocimiento de las aves fósiles de la época araucoentrerriana.. Physis.

[8] Chiappe LM, Bertelli S (2006). Skull morphology of giant terror birds.. Nature.

[9] Wroe S, Argot C, Dickman C (2004). On the rarity of big fierce carnivores and primacy of isolation and area: tracking large mammalian carnivore diversity on two isolated continents.. Proc R Soc London B.

[10] Holiday CM, Witmer LM (2008). Cranial kinesis in dinosaurs: intracranial joints, protractor muscles, and their significance for cranial evolution and function in diapsids.. J Ver Paleontol.

[11] Rayfield EJ (2005). Using finite-element analysis to investigate suture morphology: a case study using large carnivorous dinosaurs.. Anat Rec.

[12] Moazen M, Curtis N, O'Higgins P, Jones MEH, Evans SE (2009). Assessment of the role of sutures in a lizard skull: a computer modelling study.. Proc R Soc B.

[13] Bout RG, Zweers GA (2001). The role of cranial kinesis in birds.. Comp Biochem Physiol A.

[14] Gussekloo WS, Bout RG (2005). Cranial kinesis in palaeognathus birds.. J Exp Biol.

[15] Gussekloo WS, Vosselman MG, Bout RG (2001). Three-dimensional kinematics of skeletal elements in avian prokinetic and rhynchokinetic skulls determined by roentgen stereophotogrammetry.. J Exp Biol.

[16] Baumel JJ, Raikow RJ, Baumel J, King A, Breazile J, Evans H, Vanden Berge J (1993). Arthrologia.. Handbook of avian anatomy: Nomina Anatomica Avium.

[17] Zusi RL, Hanken J, Hall BK (1993). Patterns of diversity in the Avian Skull.. The Skull. Volume 2: Patterns of structural and systematic diversity.

[18] McDowell S (1948). The bony palate of birds: Part I the Palaeognathae.. Auk.

[19] Reid J (1835). Anatomical description of the Patagonian penguin.. Proc Zool Soc London.

[20] Schoonees J (1963). Some aspects of the cranial morphology of *Colius indicus*.. Annale Universiteit van Stellenbosch.

[21] Höfling E, Gasc JP (1984). Biomécanique du crâne et du bec chez *Ramphastos* (Ramphastidae, Aves).. Gegenbaurs morphologisches Jahrbuch.

[22] Sims SW (1955). The morphology f the head of the hawfinch (*Coccothraustes coccothraustes*).. Bull Brit Museum, Zool.

[23] van der Meij MAA, Bout RG (2004). Scaling of jaw muscle size and maximal bite force in finches.. J Exp Biol.

[24] van der Meij MAA, Bout RG (2006). Seed husking time and maximal bite forces in finches.. J Exp Biol.

[25] Herrel A, Podos J, Huber SK, Hendry AP (2005a). Bite performance and morphology in a population of Darwin's finches: implications for the evolution of beak shape.. Funct Ecol.

[26] Herrel A, Podos J, Huber SK, Hendry AP (2005b). Evolution of bite force in Darwin's finches: a key role for head width.. J Evol.

[27] Herrel A, Van Damme R, Vanhooydonck B, De Vree F (2001). The implications of bite performance for diet in two species of lacertid lizards.. Can J Zool.

[28] Herrel A, O'Reilly JC, Richmond AM (2002). Evolution of bite force in turtles.. J Evol Biol.

[29] Jimenez J, Jaksic F (1990). Historia natural del águila *Geranoaetus melanoleucus*: una revisión.. El Hornero.

[30] Abourachid A, Höfling E, Renous S (2005). Walking kinematics parameters in some paleognathous and neognathous neotropical birds.. Ornitología Neotropical.

[31] Wroe S, Moreno K, Clausen PD, McHenry CR (2007). High-resolution computer simulation of hominid cranial mechanics.. The Anatomical Record.

[32] McHenry CR, Wroe S, Clausen PD, Moreno K, Cunningham E (2007). Super-modeled sabercat, predatory behaviour in *Smilodon fatalis* revealed by high-resolution 3-D computer simulation.. PNAS.

[33] Preuschoft H, Witzel U (2002). Biomechanical investigations on the skulls of reptiles and mammals.. Senckenbergiana Lethaea.

[34] Metzger K, Aerts P, D'Août K, Herrel A, Van Damme R (2002). Cranial kinesis in lepidosaurs: skulls in motion.. Topics in Functional and Ecological Vertebrate Morphology.

[35] Moazen M, Curtis N, O'Higgins P, Evans SE, Fagan MJ (2009). Biomechanical assessment of evolutionary changes in the lepidosaurian skull.. PNAS.

[36] Sinclair W, Farr M, Scott W (1932). Aves of the Santa Cruz beds.. Reports of the Princeton University expeditions to Patagonia (1896–1899).

[37] Degrange FJ (2008). M. adductor mandibulae externus of *Andalgalornis steulleti* (Aves, Phorusrhacidae): Reconstruction and biomechanics (in Spanish)..

[38] Wroe S, Huber DR, Lowry M, McHenry C, Moreno K (2008). Three-dimensional computer analysis of white shark jaw mechanics: how hard can a great white bite?. J Zool.

[39] Sustaita D (2008). Musculoskeletal underpinnings to differences in killing behavior between North American Accipiters (Falconiformes: Accipitridae) and Falcons (Falconidae).. J Morphol.

[40] Wroe S, McHenry C, Thomason J (2005). Bite club: comparative bite force in big biting mammals and the prediction of predatory behaviour in fossil taxa.. Proc Biol Sci.

[41] Christiansen P, Wroe S (2007). Bite forces and evolutionary adaptations to feeding ecology in carnivores.. Ecology.

[42] Blanco RE, Jones WW (2005). Terror birds on the run: A mechanical model to estimate its maximum running speed.. Proc R Soc B.

[43] Tambussi CP (1997). Some biomechanical aspects of the phorusrhacid locomotion (Aves, Gruiformes) (in Spanish).. Ameghiniana.

[44] Patterson B, Kraglievich L (1960). Sistemática y nomenclatura de las aves fororracoideas del Plioceno Argentino.. Publicación del Museo Municipal Ciencias Naturales y Tradicionales de Mar del Plata.

[45] Campbell KE, Marcus L (1992). The relationship of hindlimb bone dimensions to body weight in birds. In: Campbell KE editors. Papers in avian paleontology honoring Pierce Brodkorb.. Natural History Museum of Los Angeles County, Science Series.

[46] Rayfield EJ (2004). Cranial mechanics and feeding in *Tyrannosaurus rex*.. Proc R Soc London B.

[47] Rayfield EJ, Norman DB, Horner CC, Horner JR, Smith PM (2001). Cranial design and function in a large theropod dinosaur.. Nature.

[48] Richmond BG, Wright BW, Grosse L, Dechow PC, Ross CF (2005). Finite element analysis in functional morphology.. Anat Rec A.

[49] Tizzard A, Horesh L, Yerworth RJ, Holder DS, Bayford RH (2005). Generating accurate finite element meshes for the forward model of the human head in EIT.. Physiol Meas.

[50] McHenry CR, Clausen PD, Daniel WJT, Meers MB, Pendharkar A (2006). The biomechanics of the rostrum in crocodilians: a comparative analysis using finite element modelling.. Anat Rec.

[51] Wroe S (2008). Cranial mechanics compared in extinct marsupial and extant African lions using a finite-element approach.. J Zool.

[52] Moreno K, Wroe S, Clausen PD, McHenry CR, D'Amore DC (2008). Cranial performance in the Komodo dragon (*Varanus komodoensis*) as revealed by high-resolution 3-D finite element analysis.. J Anat.

