# Supplementary figures and images for: Mechanical Analysis of Feeding Behavior in the Extinct “Terror Bird” Andalgalornis steulleti (Gruiformes: Phorusrhacidae)
Source: PLoS One. 2010 Aug 18;5(8):e11856. doi: 10.1371/journal.pone.0011856 (PMC2923598; doi:10.1371/journal.pone.0011856)

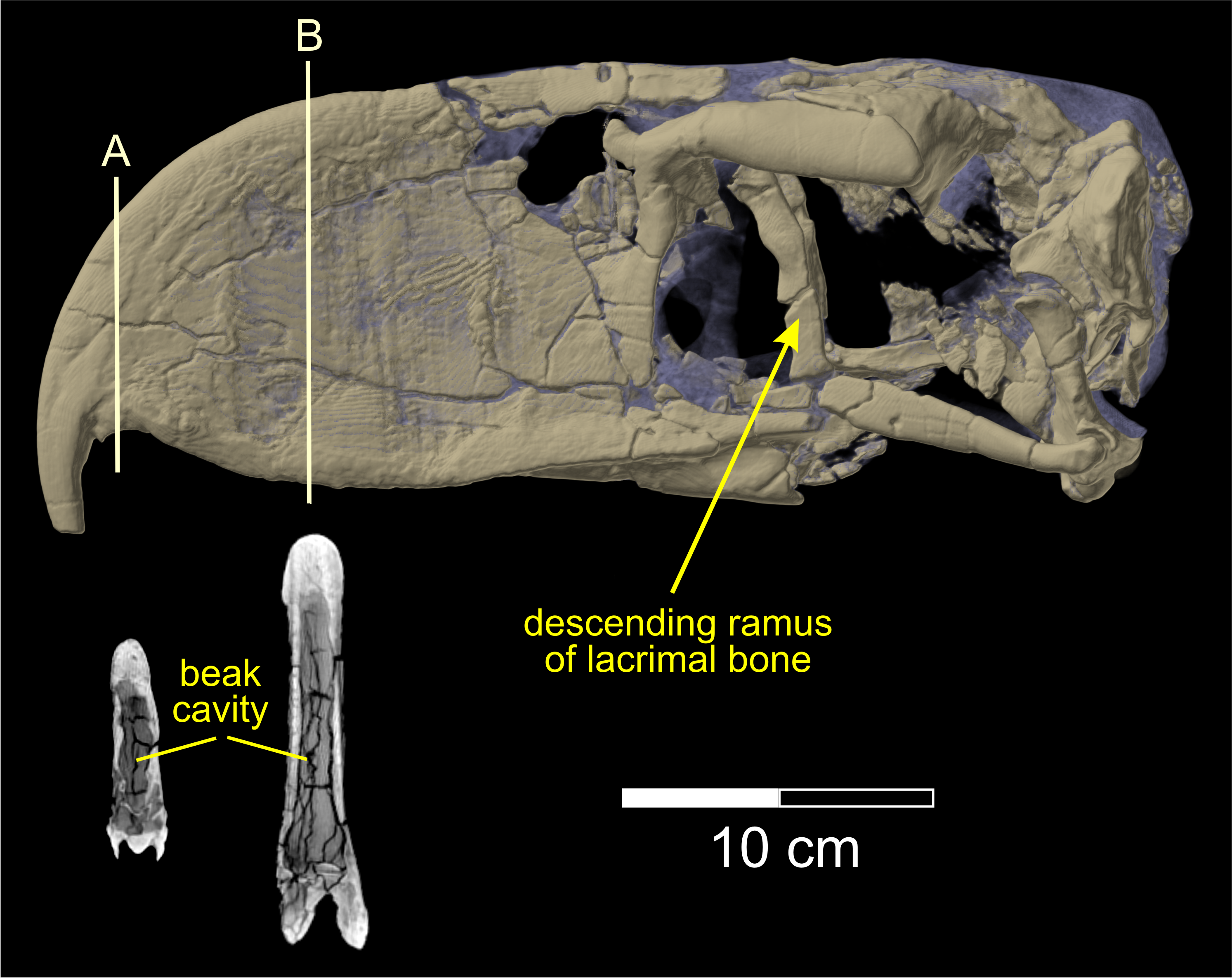

Supplement: Figure S1 — Skull of Andalgalornis steulleti (FMNH P1435). Left lateral view (volume rendering of CT scan data) with slice planes (A-B) displaying the hollow beak cavity. (3.96 MB TIF) [file pone.0011856.s001.tif]

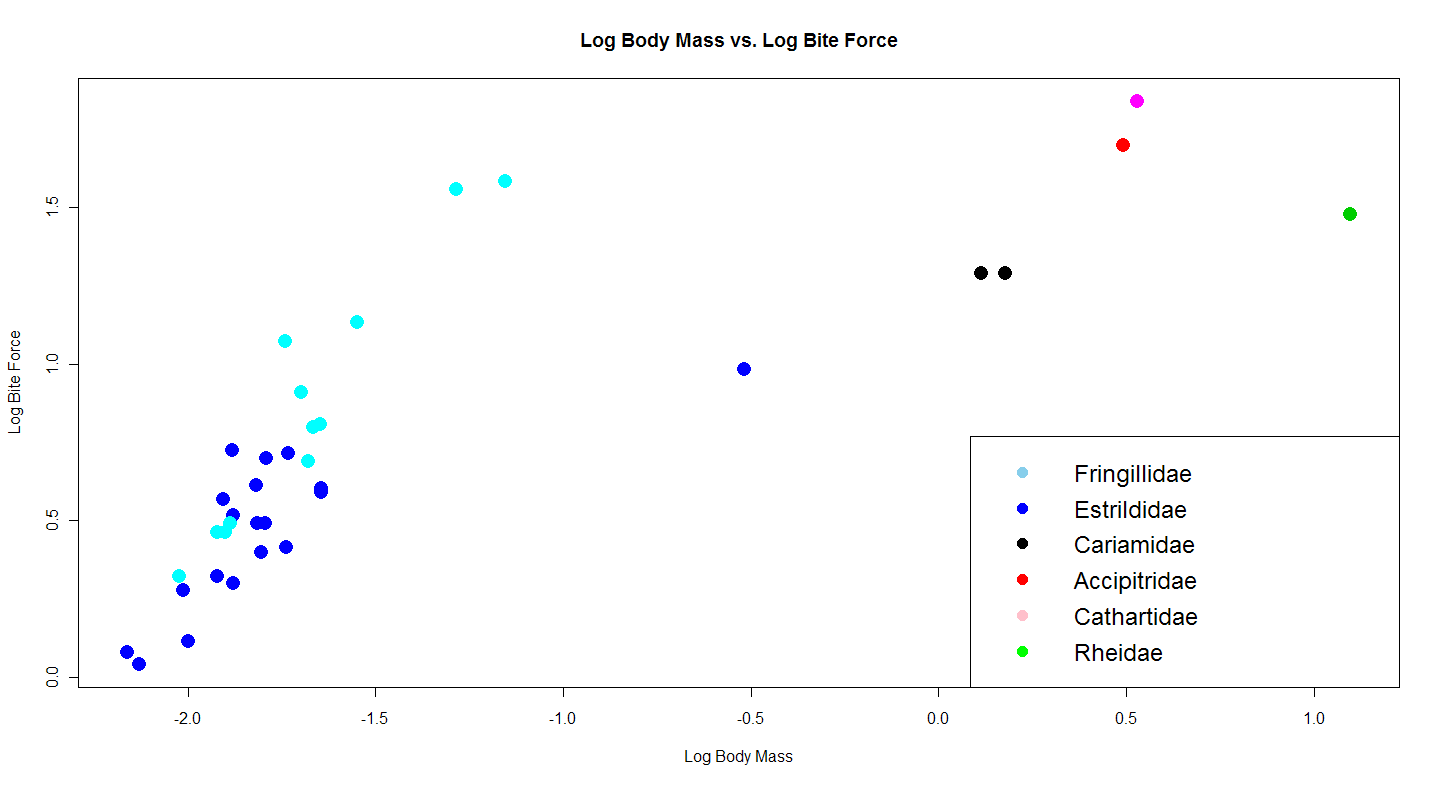

Supplement: Figure S2 — Log bite force in birds plotted against log body mass. Passeriforms in blue (dark blue: Estrildidae, sky blue: Fringillidae), Rheidae in green, Accipitridae in red, Cathartidae in rose and Cariamidae in black. See Table S1 for raw data. (3.39 MB TIF) [file pone.0011856.s002.tif]
