# Supplementary material for: Mechanical Analysis of Feeding Behavior in the Extinct “Terror Bird” Andalgalornis steulleti (Gruiformes: Phorusrhacidae)
Source: PLoS One. 2010 Aug 18;5(8):e11856. doi: 10.1371/journal.pone.0011856 (PMC2923598; doi:10.1371/journal.pone.0011856)
Supplement: Table S1 — Bite force and body mass table. (0.07 MB DOC) [file pone.0011856.s003.doc]

**Table S1.** **Bite force and body mass table**

| Species | Order | Family | BM (kg) | BF (N) |
| --- | --- | --- | --- | --- |
| *Rhea americana* | Rheiformes | Rheidae | 12.5 | 30.095 |
| *Geranoaetus melanoleucus* | Falconiformes | Accipitridae | 3.1 | 50 |
| *Sarcoramphus papa* | Ciconiiformes | Cahartidae | 3.375 | 69 |
| *Cariama cristata* | Gruiformes | Cariamidae | 1.5 | 19.42 |
| *Chunga burmeisteri* | Gruiformes | Cariamidae | 1.3 | 19.42 |
| *Amadina erythrocephala* | Passeriformes | Estrildidae | 0.0227 | 4 |
| *Amadina fasciata* | Passeriformes | Estrildidae | 0.0185 | 5.2 |
| *Chloebia gouldia* | Passeriformes | Estrildidae | 0.0152 | 4.1 |
| *Erythrura trichroa* | Passeriformes | Estrildidae | 0.0131 | 5.3 |
| *Estrilda troglodytes* | Passeriformes | Estrildidae | 0.0074 | 1.1 |
| *Hypargos niveoguttatus* | Passeriformes | Estrildidae | 0.0161 | 3.1 |
| *Lagonosticta senegala* | Passeriformes | Estrildidae | 0.0069 | 1.2 |
| *Lonchura fringilloides* | Passeriformes | Estrildidae | 0.0162 | 5 |
| *Lonchura pallida* | Passeriformes | Estrildidae | 0.0132 | 3.3 |
| *Lonchura punctulata* | Passeriformes | Estrildidae | 0.0124 | 3.7 |
| *Neochima modesta* | Passeriformes | Estrildidae | 0.0132 | 2 |
| *Neochmia ruficauda* | Passeriformes | Estrildidae | 0.012 | 2.1 |
| *Padda oryzivora* | Passeriformes | Estrildidae | 0.304 | 9.6 |
| *Phoephila acuticauda* | Passeriformes | Estrildidae | 0.0183 | 2.6 |
| *Taeniopygia bichenovi* | Passeriformes | Estrildidae | 0.0097 | 1.9 |
| *Poephila cincta* | Passeriformes | Estrildidae | 0.0157 | 2.5 |
| *Pytilia hypogrammica* | Passeriformes | Estrildidae | 0.0153 | 3.1 |
| *Taenopygia guttata* | Passeriformes | Estrildidae | 0.0227 | 3.9 |
| *Uraeginthus bengalus* | Passeriformes | Estrildidae | 0.01 | 1.3 |
| *Carduelis chloris* | Passeriformes | Fringillidae | 0.0283 | 13.6 |
| *Carduelis flammea* | Passeriformes | Fringillidae | 0.0126 | 2.9 |
| *Carduelis sinica* | Passeriformes | Fringillidae | 0.02 | 8.1 |
| *Carduelis spinus* | Passeriformes | Fringillidae | 0.013 | 3.1 |
| *Carpodactus erythrinus* | Passeriformes | Fringillidae | 0.0216 | 6.3 |
| *Eophona migratoria* | Passeriformes | Fringillidae | 0.052 | 36.1 |
| *Mycerobas affinis* | Passeriformes | Fringillidae | 0.07 | 38.4 |
| *Pyrrhula pyrrhula* | Passeriformes | Fringillidae | 0.0209 | 4.9 |
| *Rhodopechys obsoleta* | Passeriformes | Fringillidae | 0.0225 | 6.4 |
| *Serinus leucopygius* | Passeriformes | Fringillidae | 0.0095 | 2.1 |
| *Serinus mozambicus* | Passeriformes | Fringillidae | 0.012 | 2.9 |
| *Serinus sulphuratus* | Passeriformes | Fringillidae | 0.0182 | 11.8 |

BM = Body Mass expressed in kilograms; BF = Bite Force expressed in newtons.
