# Supplementary material for: Mechanical Analysis of Feeding Behavior in the Extinct “Terror Bird” Andalgalornis steulleti (Gruiformes: Phorusrhacidae)
Source: PLoS One. 2010 Aug 18;5(8):e11856. doi: 10.1371/journal.pone.0011856 (PMC2923598; doi:10.1371/journal.pone.0011856)
Supplement: Text S1 — Andalgalornis' fossil. (0.03 MB DOC) [file pone.0011856.s004.doc]

**Text S1.** *Andalgalornis*’ fossil

*Andalgalornis steulleti* (= *A. ferox* [1]) FMNH P14357: Skull and jaws; pre-synsacral vertebral series complete except for the atlas; sixth rib complete; first, third, and fourth rib almost complete and the others with the exception of the second and the eighth are represented only by the proximal parts; pelvis and synsacrum complete.

Horizon and locality: Andalhuala Formation (~ 5 Ma, lower Pliocene), Chiquimil, Catamarca province, Argentina.

General skull morphology: Undoubtedly, the most outstanding feature of the skull is the huge beak, which in *Andalgalornis* is three times taller than wide, completely hollow (Figure S1), ends in a massive hook, and has small narial openings displaced caudodorsally. *Andalgalornis* has a relative small encephalic cavity relative to the rest of the skull. The occipital region is broad and vertical in disposition and the temporal fossa is wide, deep, and triangular. The quadrate is massive and obliquely positioned such that the jaw joint is retracted caudally beyond the level of the occiput. On the contrary, in extant birds the quadrate is directed forward, and the mandibular articulation is situated more rostrally, in front of the occiput. The lacrimal is well developed and it is fused anteriorly with the nasals and frontals. It shows two very robust rami: one forms the supraorbital lamina and the other one descends, contacting the jugal bar by a wide area. There is a little medial flange in the area of the jugal contact that makes the articulation even stiffer. Hypertrophy of this descending ramus seems to be a found only in the larger phorusrhacids (e.g., the patagornithine *Patagornis*, the phorusrhacine *Kelenken*, but not in the Psilopterinae). Nevertheless, the homology of this descending ramus has not been fully tested. In the seriemas, the descending ramus of the lacrimal is well developed but it reaches the jugal bar through a rod-like bone (Burmesiter in [2]). This bone seems to be the ossification of the *lig. lacrimojugale*. It seems that the descending ramus of the lacrimal in *Andalgalornis* (and the larger phorusrhacids) is formed by the fusion of the descending ramus itself and this ossification of the *lig. lacrimojugale*. This feature has not been observed in other birds.

1. Patterson B, Kraglievich L (1960) Sistemática y nomenclatura de las aves fororracoideas del Plioceno Argentino. Publicación del Museo Municipal Ciencias Naturales y Tradicionales de Mar del Plata 1: 1-51.

2. Andrews CW (1896) Remarks on the Stereornithes, a group of extinct birds from Patagonia. Ibis 7 (2): 6-12.
